# Infantile hepatic hemangioma misdiagnosed by prenatal ultrasonography: A case report

**DOI:** 10.1097/MD.0000000000024242

**Published:** 2021-01-15

**Authors:** Ya Jin, Lin Li, Fan Yang

**Affiliations:** a Department of Ultrasound; b Key Laboratory of Obstetrics and Gynecology and Pediatric Diseases and Birth Defects of Ministry of Education, West China Second Hospital of Sichuan University; c Department of Pathology, West China Hospital of Sichuan University; d Chengdu Chenghua District Maternal and Child Health Hospital, Chengdu, Sichuan, China.

**Keywords:** differential diagnosis, fetus, hepatoblastoma, infantile hepatic hemangioma, ultrasound

## Abstract

**Rationale:**

The drastic differences in treatment and prognosis of infantile hepatic hemangioma (IHH) and hepatoblastoma (HBL) make accurate prenatal diagnosis imperative. The retrospective comparisons of ultrasonic features between fetal IHH and HBL have been reported before, but clinically, the differential diagnosis in utero is very difficult and can lead to prenatal misdiagnosis.

**Patient concerns:**

A 27-year-old woman at 30 gestational weeks underwent the routine prenatal examination. A heterogeneous solid mass of the fetus, with close relationship to the liver, was recognized by ultrasound.

**Diagnosis:**

A diagnosis of HBL was highly considered.

**Interventions:**

The fetus was aborted and the autopsy was performed.

**Outcomes:**

The histological outcome was IHH.

**Lessons:**

The prognosis of fetal IHH and HBL is very different, so an accurate diagnosis prenatally is crucial and indispensable. The radiologist and clinician should differentiate between IHH and HBL, especially since the fetus can have serious complications.

## 1 Introduction

Infantile hepatic hemangioma (IHH) is the most common benign mesenchymal tumor of the liver in fetus and infants, accounting for about 20% of all liver tumors arising in fetus,^[1,2]^ while hepatoblastoma (HBL) is the most frequent malignant congenital neoplasm.^[3]^ Giant IHH may cause life-threatening complications in utero, including fetal hydrops, anemia, thrombocytopenia, cardiac failure. IHH could regress spontaneously without treatment. The core treatment for IHH is conservative therapy (observation or follow-up).^[4,5]^ However, the mainstay treatment for HBL are surgical resection, neoadjuvant chemotherapy, and even liver transplantation, and the prognosis depends on many factors and is not encouraging.^[6]^

Since there exist differences in the treatment therapy and prognosis between IHH and HBL, how to make an accurate prenatal differential diagnosis is an issue. The ultrasound has become the main method for prenatal diagnosis, owing to its real-time assessment of the liver tumor in fetus, including size, location, the feeding vessels, even the relationship between portal and hepatic vein.^[7]^ However, the overlap of imaging features between IHH and HBL may confuse the differential diagnosis in utero, and there is very few research about the prenatal differential diagnosis.^[8]^ The final diagnosis depends on the postnatal ultrasound, contrast-enhanced computerized tomography and the biopsy of the hepatic lesions.^[9]^

In this case report and the review of literature, we tried to investigate if there were specific imaging features of ultrasound for the differential diagnosis between IHH and HBL in utero.

## 2 Case presentation

A 27-year-old woman, gravida 1 para 0, underwent the routine prenatal examinations in our hospital from 6 gestational weeks. Till 30 gestational weeks, the prenatal course was unremarkable, including systemic prenatal ultrasound diagnosis and the routine fetal cardiac screening (at 24 gestational weeks). Fetal growth was within the normal process and the alpha fetal protein was 23.91 U/ml (the normal reference value:0–25 U/ml).

**Figure 1:**
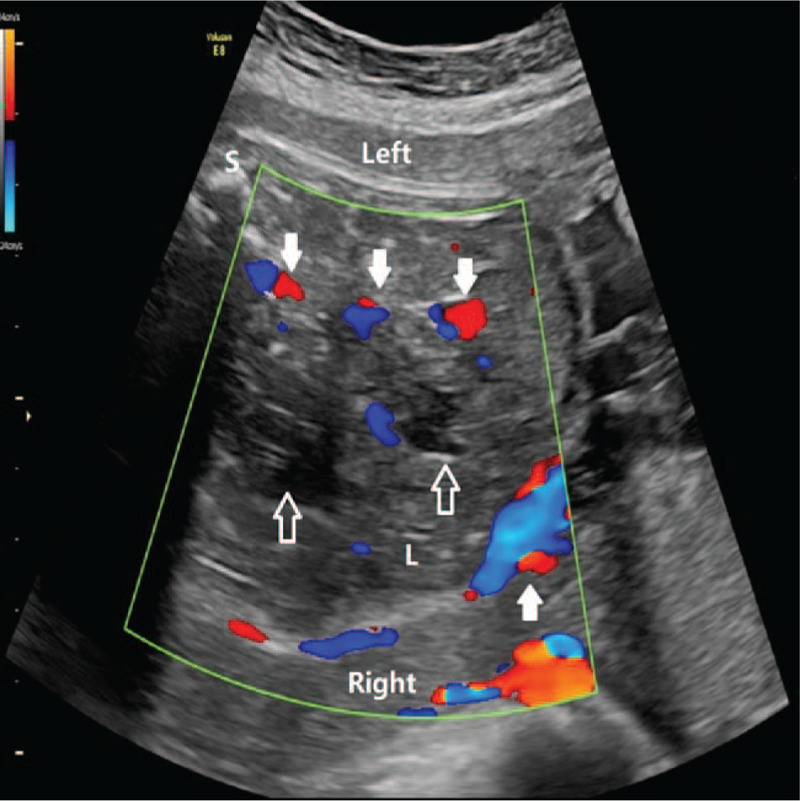
Ultrasonic features in B-mode and color Doppler. A heterogeneous solid mass located in the right quadrant of the fetal abdominal cavity, with close relationship to the liver. The lesion seemed to have ill-defined margin, irregular shape, partial capsule and multiple cystic cavities inside (hollow arrow). The mass appeared to be highly vascularized (solid arrow). (s: spine, L: liver, Left: the left side; Right: the right side).

**Figure 2:**
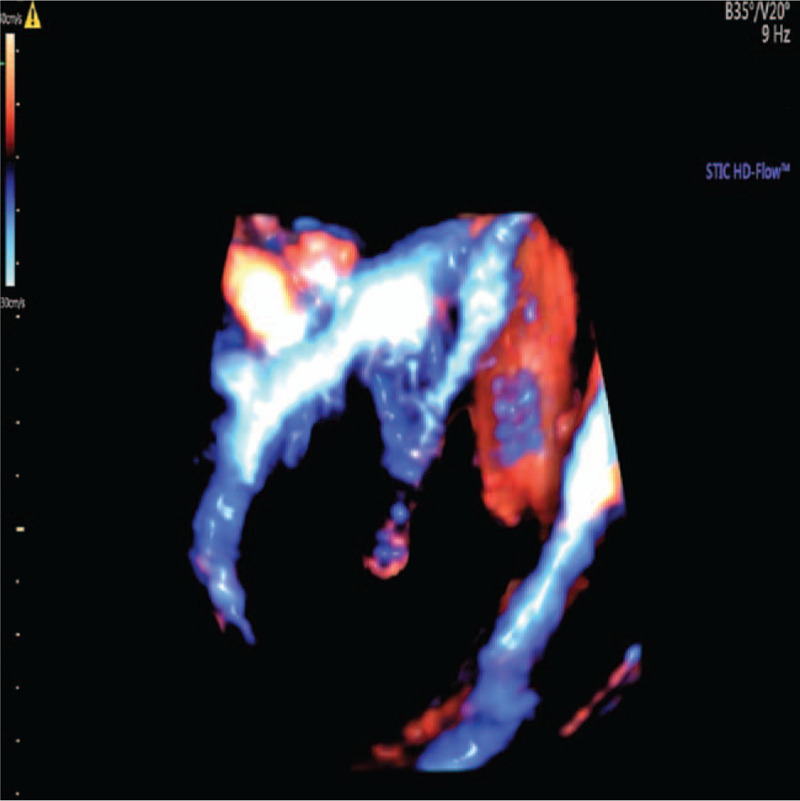
Ultrasonic features in power Doppler. The mass appeared to be highly vascularized around the lesion but with less vessels inside (dark part in center).

At 30 gestational weeks, the B-mode ultrasound revealed that the liver of the fetus was enlarged with a heterogeneous solid mass located in the right quadrant of the fetal abdominal cavity, with a close relationship to the liver. The mass measured 5.8cmx4.7cmx5.2 cm and pushed the portal vein and gall bladder. The lesion seemed to have an ill-defined margin, irregular shape, partial capsule with multiple cystic cavities inside, and maximal diameter of 2.1 cm. The portal and hepatic veins were not dilated. In the color Doppler ultrasound, the mass appeared to be highly vascularized (Fig. 1) and RI was 0.67. The three-dimensional power Doppler ultrasound showed the feeding vessels were mainly around the lesion but there were less vessels inside it (Fig. 2). Due to the rapid enlargement of this mass and the imaging features of ultrasound, the diagnosis of HBL was highly considered. But IHH, mesenchymal hamartoma or other rare liver tumors such as hepatic cysts or adenomas could not be ruled out.

According to the pediatrician, the final diagnosis depended on the postnatal biopsy of the hepatic lesion and the prognosis of HBL was uncertain. Finally, the parents chose to give up this fetus because of the uncertain outcomes involving tremendous emotional and economic impacts on the family. Furthermore, they refused to take any further prenatal examination, like fetal MRI. A male fetus was aborted, weighing 1409 g and measuring 36 cm long.

**Figure 3:**
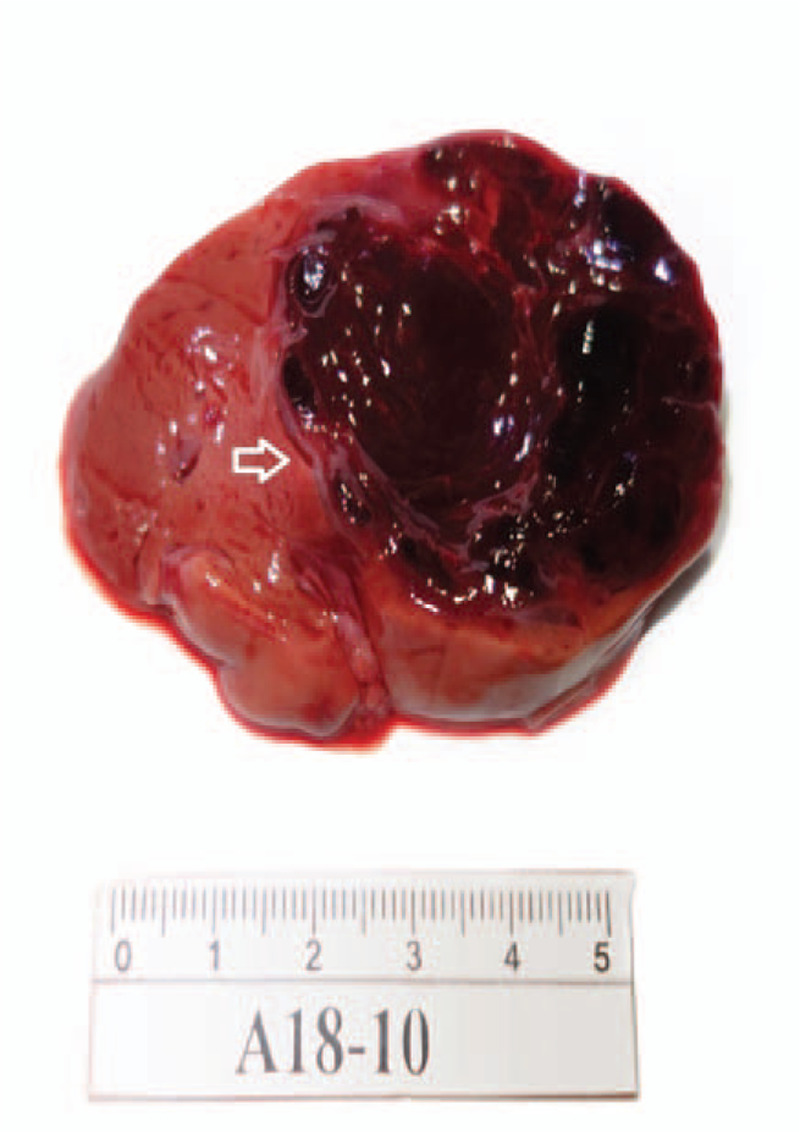
Gross specimen. During the autopsy, the liver of the fetus was enlarged, about 8 centimeters long and the mass was located in the right lobe, pushing the portal vein (The dark part is this tumor).

**Figure 4:**
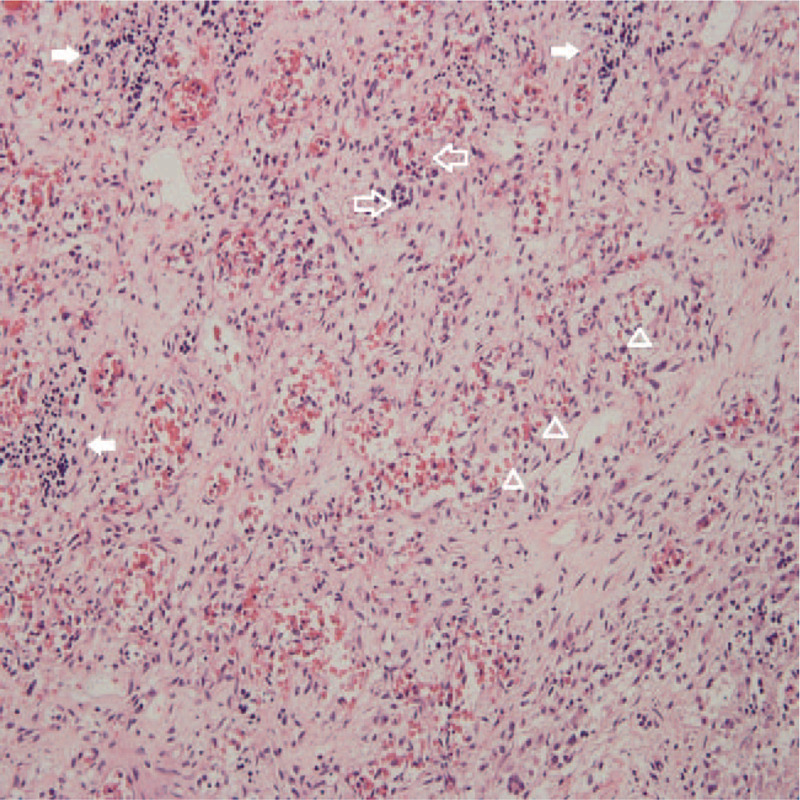
HE staining pathological section. In the hematoxylin and eosin stain (HE, ×200), the tumor was composed of a large number of small vessels with thin wall. The vascular endothelial cells were large and monolayered. The extramedullary hematopoiesis was observed.

Medical ethics committee of West China Second Hospital of Sichuan University have approved this case report and the autopsy was performed. The liver of the fetus was enlarged, about 8 cm long and the mass was located in the right lobe, pushing the portal vein (Fig. 3). The histological diagnosis was IHH (Fig. 4).

The written consent was obtained from the patient for publication of case details and images.

## 3 Discussion

Most IHH and HBL share similar clinical symptoms in utero and the imaging findings combined with the level of alpha fetal protein play a vital role for the diagnosis and treatment strategies selection.^[10,11]^ Ultrasound is the routine scan method during the prenatal diagnosis of IHH and HBL. However, the overlap of ultrasonic imaging between them bring difficulties in the prenatal differential diagnosis. In this case report, we aim to analyze the reasons for misdiagnosis and to investigate if there were specific ultrasonic imaging features for the differentiation between IHH and HBL in utero.

In this case report, the B-mode ultrasound revealed a heterogeneous solid mass with multiple cystic cavities inside the fetus. These imaging features were similar to IHH of previous studies.^[12]^ IHH may be detected as early as 16 weeks using US, and has an appearance of a complex echo pattern on gray-scale sonograms, heterogeneous, mostly hypoechoic. According to the study of Belinda et al,^[3]^ giant hepatic hemangiomas appeared as heterogeneous with a central necrotic hypoechoic area inside. However, HBL could also appear as a heterogeneous, mostly hypoechoic solid mass with foci of hemorrhage and necrosis inside.^[9,12,13,14]^ This was one reason for the misdiagnosis in our case. The other reason is that IHH contains arteriovenous malformations and venous, lymphatic or capillary components thus cystic cavity inside is regularly seen and this important feature was ignored by the sonographer.^[3,8,15,16]^ In addition, the lesion seemed to have an ill-defined margin, irregular shape and partial capsula and these appearances were consistent with HBL.^[17,18]^

In the color Doppler ultrasound, studies showed that HBL appeared as arterial flow with RI > 0.7 and it may be that HBL mainly consists of numerous and disorderly hepatoblast-like cells and lack of arterio-venous anastomosis or draining veins.^[19–21]^ Studies also showed that IHH appeared to have large feeding and draining vessels surrounding or within the tumors and the presence of artery-vein shunting. IHH was supplied by hepatic artery and drained by hepatic vein. Both vessels were enlarged and showed high velocity and low RI (<0.7).^[7,19–21]^ This could be significant imaging finding specific to IHH. However, in this case, the mass appeared to be a highly vascularized lesion, RI was 0.67 but the portal and hepatic veins of the fetus were not dilated, and according to the power Doppler ultrasound, the feeding vessels of the lesion was unclear. This is another reason for the misdiagnosis in our case.

There exist difficulties in the differential diagnosis between IHH and HBL in utero by ultrasound. But according to some specific imaging features like necrosis or cystic cavity inside the tumor, ill-defined margin, polylobular, the type of the feeding vessels (arteries or veins), dilated hepatic arteries, veins, or artery-vein shunting, IHH could be diagnosed more accurately prenatally.

## Author contributions

**Conceptualization:** Ya Jin, Fan Yang.

**Data curation:** Ya Jin, Fan Yang.

**Formal analysis:** Ya Jin, Fan Yang.

**Funding acquisition:** Fan Yang.

**Methodology:** Ya Jin, Fan Yang.

**Project administration:** Fan Yang.

**Resources:** Lin Li.

**Validation:** Lin Li.

**Visualization:** Lin Li.

**Writing – original draft:** Ya Jin.

**Writing – review & editing:** Fan Yang.

## Abbreviations

Abbreviations: HBL = hepatoblastoma, HE = hematoxylin and eosin stain, IHH = infantile hepatic hemangioma, RI = resistance index.
